# Bergapten drives autophagy through the up-regulation of PTEN expression in breast cancer cells

**DOI:** 10.1186/s12943-015-0403-4

**Published:** 2015-07-07

**Authors:** Francesca De Amicis, Saveria Aquila, Catia Morelli, Carmela Guido, Marta Santoro, Ida Perrotta, Loredana Mauro, Francesca Giordano, Alessandra Nigro, Sebastiano Andò, Maria L. Panno

**Affiliations:** Department of Pharmacy, Health and Nutritional Sciences, University of Calabria, 87036 Arcavacata di Rende, Cosenza Italy; Health Center, University of Calabria, 87036 Arcavacata di Rende, Cosenza Italy; Department of Biology, Ecology and Earth Science (Di.B.E.S.T.), University of Calabria, 87036 Arcavacata di Rende, Cosenza Italy

**Keywords:** Cell survival, Psoralen, NF-Y, PTEN, AKT

## Abstract

**Background:**

Bergapten (5-methoxypsoralen), a natural psoralen derivative present in many fruits and vegetables, has shown antitumoral effects in a variety of cell types. In this study, it has been addressed how Bergapten in breast cancer cells induces autophagic process.

**Results:**

In MCF7 and ZR-75 breast cancer cells Bergapten exhibited anti-survival response by inducing the autophagic process increasing Beclin1, PI3KIII, UVRAG, AMBRA expression and conversion of LC3-I to LC3-II. LC3-GFP, Acridine orange assay and transmission electron microscopy even confirmed the increased autophagosome formations in treated cells. Bergapten-induced autophagy is dependent by PTEN up-regulation, since silencing this gene, the induction of Beclin1 and the p-AKT/p-mTOR signal down-regulation were reversed. PTEN is transcriptionally regulated by Bergapten through the involvement of p38MAPK/NF-Y, as evidenced by the use of p38MAPK inhibitor SB203580, site-direct mutagenesis of NF-Y element and NF-Y siRNA. Furthermore NF-Y knockdown prevented Bergapten-induced acid vesicular organelle accumulations (AVOs), strengthening the role of this element in mediating autophagy.

**Conclusions:**

Our data indicate PTEN as a key target of Bergapten action in breast cancer cells for the induction of autophagy. These findings add further details on the mechanism of action of Bergapten, therefore suggesting that phytochemical compounds may be implemented in the novel strategies for breast cancer treatment.

**Electronic supplementary material:**

The online version of this article (doi:10.1186/s12943-015-0403-4) contains supplementary material, which is available to authorized users.

## Background

Breast cancer is the most frequent malignant neoplasm of female in Europe and North America and breast cancerogenesis is still not fully recognized, due to many risk factors acting in a dynamic bio-molecular context. Besides, prognostic factors commonly used for the follow up of patients evidence an incomplete picture of the breast tumor biology [1].

The oncosuppressor gene PTEN (Phosphatase tensin homologue deleted from chromosome 10) is often mutated in hormone-related tumors among women, including breast [2] and PTEN expression correlates negatively with neoplasm advancement grade [3]. PTEN codes protein/lipid phosphatase governing phosphatidylinositol 3-kinase (PI3K) signaling thus contributing to the control of the proliferation, differentiation and apoptotic process [4]. PTEN has also been shown to mediate autophagy in mammalian cells through its lipid phosphatase activity which antagonizes the inhibitory effect of the PI3K/AKT pathway on the autophagic sequestration that involves type III PI3-kinase [5].

The substantial evidence that the progression of breast cancer can be influenced by PI3K/AKT/mTOR, suggests the use of specific inhibitors as component of therapeutic portfolio that could be able to increase the expression of oncosuppressor PTEN.

In recent years, many naturally occurring compounds, sometimes present in the diet have gained considerable attention as antitumor agents [6, 7]. In this regard cumarine-derivate compound 5-methoxypsoralen (Bergapten, Bg) has been shown to be antiproliferative and protective against various types of breast cancer cells [8, 9], although limited information of molecular mechanism of action is still available.

In particular, photo-activation of psoralen with UVA irradiation, used in the treatment of proliferative skin disorders, exerts antitumor effects in models of human breast cancer that overexpress the ErbB2 receptor tyrosine kinase oncogene, through a mechanism mediated by inhibition of ErbB2 signaling [10]. Furthermore, we recently reported that Bg, independently by its photo-activation, generates membrane signaling pathways able to address apoptotic responses in breast cancer cells [9]. Besides, the Bg in Tamoxifen-sensitive and Tamoxifen-resistant breast cancer cells can act as estrogen receptor alpha down-regulator, through a process involving the SMAD4 protein [8].

In the present study we provide evidence that Bg evokes an autophagic phenotype associated with an anti-survival response in breast cancer cells. These effects are mediated by the up-regulation of PTEN expression due to the activation of p38MAPK/NF-Y signaling.

## Results

### Bergapten upregulates PTEN expression and PTEN gene promoter activity in MCF-7 and ZR-75 breast cancer cells

The PI3K is one of the most important pathway in cancer cells for the integration of different functions, including cellular metabolism, differentiation, and survival. We have previously reported [11] that Bg was able to decrease the PI3K activity, thus addressing apoptotic responses in breast cancer cells. Based on these promising initial data, we investigated if the drug was capable to influence the PI3K inhibitor PTEN and its downstream effector Akt.

**Fig. 1:**
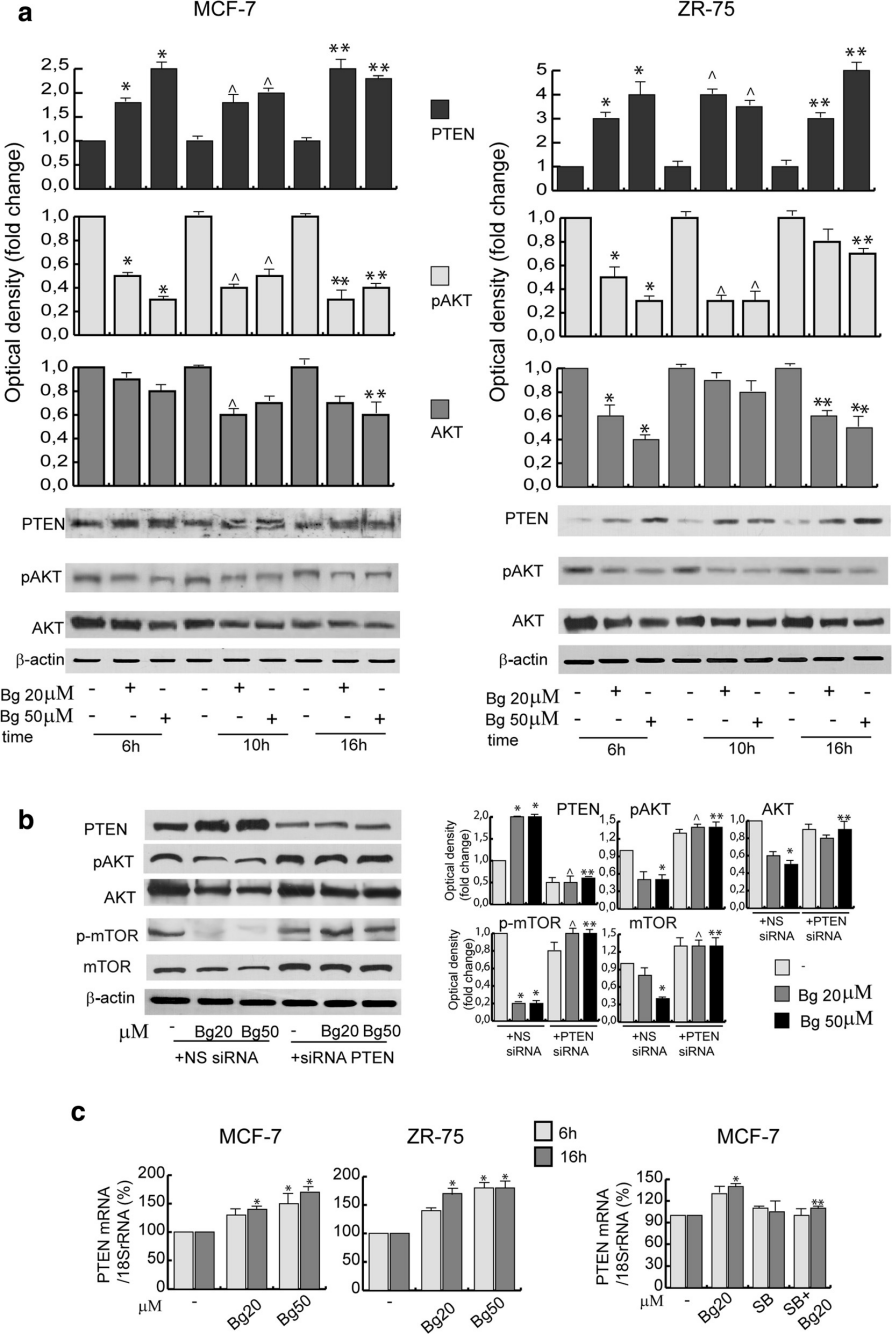
Bergapten-regulates PTEN protein and mRNA levels in breast cancer cells. **a** Western blot analysis of PTEN, p-AKT and AKT in MCF-7 and ZR-75 breast cancer cells treated with vehicle (−) or Bg 20 μM or Bg 50 μM at different times as indicated. β-actin was used as loading control. Autoradiographs show the results of one representative experiment. Columns, are mean of three independent experiments in which band intensities were evaluated in terms of optical density arbitrary units and expressed as fold change over vehicle, which was assumed to be 1; bars, SD;* *P* < 0.05 vs 6 h vehicle. ^ *P* < 0.05 vs 10 h vehicle. ** *P* < 0.05 vs 16 h vehicle. **b** Western blot analysis of PTEN, p-AKT, AKT, p-mTOR and mTOR in MCF-7 cells transfected with non specific (NS) or targeted against PTEN siRNA treated with vehicle (−) or Bg 20 μM or Bg 50 μM for 16 h. Autoradiographs show the results of one representative experiment. Columns, are mean of three independent experiments in which band intensities were evaluated in terms of optical density arbitrary units and expressed as fold over vehicle, which was assumed to be 1; bars, SD;* *P* < 0.05 vs NS siRNA vehicle. ^ *P* < 0.05 vs NS siRNA Bg 20 μM. ** *P* < 0.05 vs NS siRNA Bg 50 μM. **c** Real-time PCR assay of PTEN mRNA expression in MCF-7 and ZR-75 cells treated with vehicle (−), Bg 20 μM, Bg 50 μM (*left panel*) and/or p38MAPK inhibitor SB203580 (SB 10 μM) (*right panel*). 18S rRNA was determined as control. Columns are the mean of three independent experiments each in triplicate; bars, SD; *P < 0.05 vs vehicle treated cells. ** *P* < 0.05 vs Bg 20 μM

As shown in the Fig. 1, Bg treatment, from 6 to 16 h (h), induces a significant increase of PTEN expression in terms of protein (Fig. 1a, b) and mRNA content (Fig. 1c) in both MCF-7 and ZR-75 cells. As presumed, pAkt, AKT and p-mTOR, m-TOR expression levels were reduced by Bg treatment and this effect was specifically mediated by PTEN. Indeed, addition of a PTEN-targeting siRNA which resulted in reduction of the correspondent protein levels (Fig. 1b), clearly counteracted the Bg -dependent down-regulation, addressing the involvement of the oncosuppressor protein in mediating Bg effects.

**Fig. 2:**
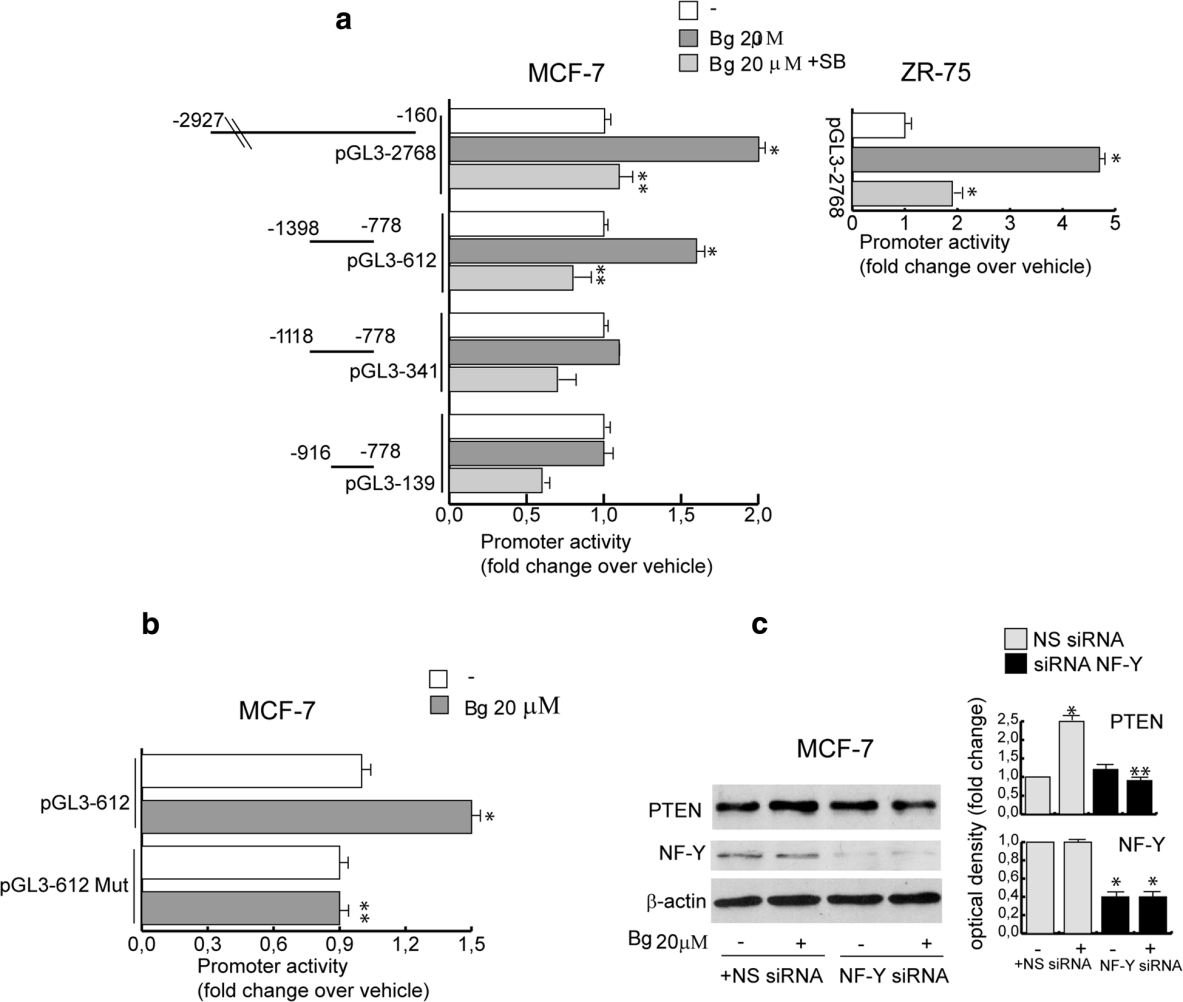
Bergapten transactivates PTEN promoter gene in MCF-7 and ZR-75 cells. **a** Left panel: schematic representation of deletion fragments of the PTEN gene promoter. Right panel: constructs depicted were transiently transfected in MCF-7 and ZR-75 cells as indicated, treated with vehicle (−), Bg 20 μM or Bg 20 μM + SB203580 (10 μM). **b** Site-directed mutagenesis of the NF-Y site. pGL3-612 and pGL3-612 Mut promoter constructs were transfected into MCF-7 cells, and promoter activity was assessed in the absence or presence of Bg 20 μM treatment. Columns are mean of three independent experiments and expressed as fold change over vehicle, which was assumed to be 1; bars, SD; *P < 0.05 vs vehicle. ** *P* < 0.05 vs pGL3-612 Bg 20 μM. **c** Western blot analysis of PTEN and NF-Y in MCF-7 cells transfected with non specific (NS) or targeted against NF-Y siRNA treated with vehicle (−) or Bg 20 μM for 16 h. β-actin was used as loading control. Autoradiographs show the results of one representative experiment out of three. Columns, are mean of three independent experiments in which band intensities were evaluated in terms of optical density arbitrary units and expressed as fold over vehicle, which was assumed to be 1; bars, SD;* *P* < 0.05 vs NS siRNA vehicle. ** *P* < 0.05 vs NS siRNA Bg 20 μM

To further investigate the molecular basis for regulation of PTEN gene expression by Bg, we transfected the full length of the PTEN promoter gene spanning from −2927 to −160 bp (pGL3-2768) previously described [12, 13] into MCF-7 and ZR-75 cells. Interestingly, the results in Fig. 2a*right panel* indicate that 20 μM Bg significantly trans-activated the pGL3-2768 construct in both cell types.

In order to identify the region of PTEN promoter responsible for Bg –induced transactivation we analyzed the activity of a series of PTEN promoter deleted constructs (Fig. 2a, *left panel*) previously described as an useful tool to study the regulation of PTEN expression [12, 13]. As revealed in Fig. 2a the promoter transactivation upon Bg was observed only in the deleted construct pGl3-612 indicating that the region between −1398 to −1118 bp was necessary for the up-regulation of PTEN promoter activity produced by Bg stimulation. Sequence analysis, showed that this region contains DNA motif able to bind NF-Y, that accounts for promoter responsiveness to Bg [9]. Since NF-Y transcription factor is a potential effector of Bg action [9] which induces p38MAPK activation as we observed in Additional file 1: Figure S1A, we wondered whether PTEN up-regulation, might be dependent of this phosphorylative pathway. In MCF-7 cells, pre-treated for 2 h with the p38MAPK inhibitor SB203580 (10 μM), known to block the kinase activities of p38 isoforms, the Bg-induced up-regulation of PTEN mRNA (Fig. 1c), protein levels (Additional file 1: Figure S1B) and gene promoter activity (Fig. 2a) were abrogated.

To further assess NF-Y involvement in the Bg -dependent induction of PTEN gene activity, we performed site directed mutagenesis to alter NF-Y motif. As shown in Fig. 2b, the activity of the mutated construct was unaffected by Bg treatment. Besides specific NF-Y siRNA substantially counteracted Bg effects on PTEN expression (Fig. 2c) addressing how this factor is fundamental for Bg action.

### Bergapten-action in breast cancer cells induces the autophagic process

**Fig. 3:**
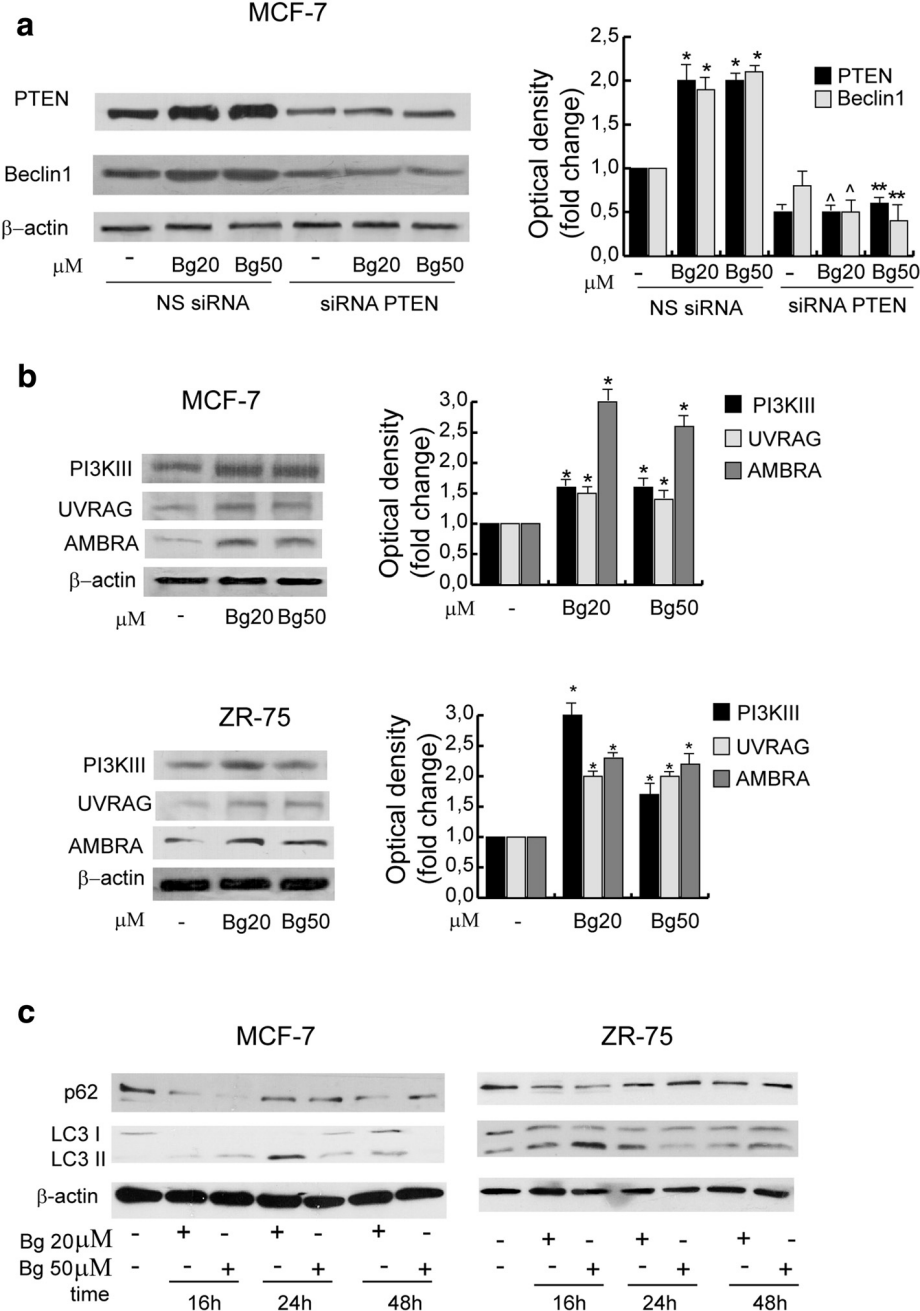
Bergapten modulates the expression of proteins involved in autophagy process in MCF-7 and ZR-75 cells. **a** Western blot analysis of PTEN (also showed in Fig. 1b) and Beclin1 in MCF-7 cells transfected with non specific (NS) or targeted against PTEN siRNA treated with vehicle (−), Bg 20 μM or Bg 50 μM for 16 h. β-actin was used as loading control. Autoradiographs show the results of one representative experiment. Columns, are mean of three independent experiments in which band intensities were evaluated in terms of optical density arbitrary units and expressed as fold change over vehicle, which was assumed to be 1; bars, SD;* *P* < 0.05 vs NS siRNA vehicle. ^ *P* < 0.05 vs NS siRNA Bg 20 μM. ** *P* < 0.05 vs NS siRNA μM. **b** Western blot analysis of PI3KIII, UVRAG and AMBRA expression in MCF-7 and ZR-75 cells treated for 16 h with vehicle (−), Bg 20 μM, Bg 50 μM. Columns, are mean of three independent experiments in which band intensities were evaluated in terms of optical density arbitrary units and expressed as fold over vehicle, which was assumed to be 1; bars, SD; *P < 0.05 vs vehicle treated cells. **c** Time course study. Western blot analysis of p62 and LC3 expression in MCF-7 and ZR-75 cells treated for different times as indicated with vehicle (−), Bg 20 μM, Bg 50 μM. Autoradiographs show the results of one representative experiment out of three

Since PTEN through inhibition of PI3K/Akt pathway modulates autophagy, we next investigated the Bg -action on the expression of the specific autophagy master regulator Beclin1, having a prevalent role in the early phases of the process. As shown in Fig. 3a Bg treatment increased Beclin1 levels and this effect was specifically mediated by PTEN, indeed in MCF-7 cells transfected with a specific PTEN-siRNA, Bg was no longer able to induce the up-regulation of Beclin1 expression. In both breast cancer cell types, Bg induced the expression of other strictly related proteins such as PI3KIII, UVRAG, and AMBRA which cooperate to the autophagosome formation (Fig. 3b).

When autophagy is activated, PI3KIII causes lipidation of LC3-I (microtubule-associated protein 1 light chain 3) which alters LC3-I electrophoretic mobility referred as LC3-II [14]. Treatment of MCF-7 and ZR-75 cells with Bg resulted in the conversion of LC3-I to LC3-II after 16 h which persisted until 24 h under Bg 20 μΜ and not more evident at 48 h (Fig. 3c). Accordingly, treatment with Bg for 16 h resulted in a transient decrease in levels of autophagic cargo receptor p62, marker of degradation via the lysosomes during autophagy.

### LC3-GFP, acridine orange assay and transmission electron microscopy (TEM) revealed formation of autophagosomes in Bergapten-treated breast cancer cells

**Fig. 4:**
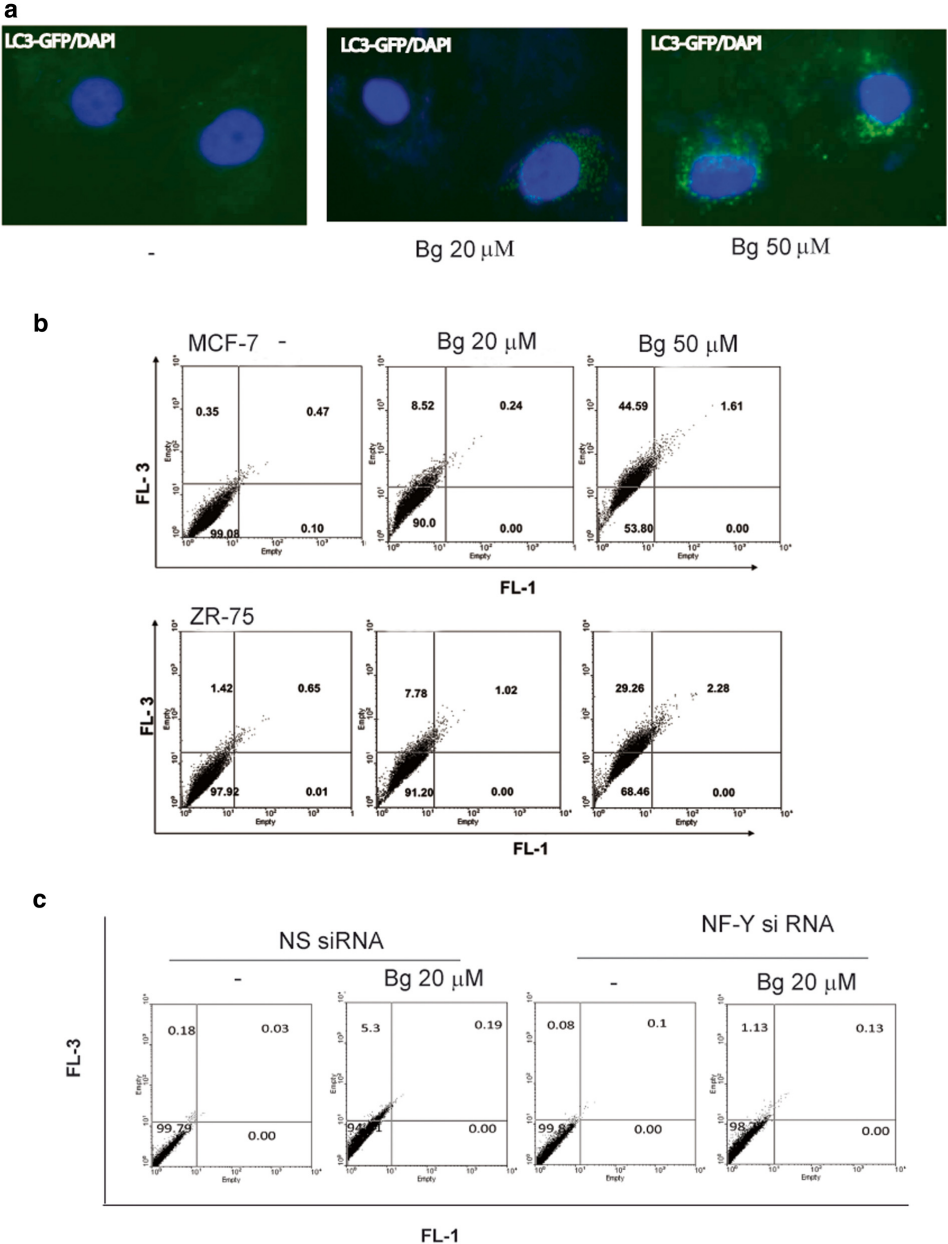
Bergapten treatement increases autophagosome formation in MCF-7 and ZR-75 cells. **a** MCF-7 cells were transiently transfected with LC3-GFP and treated with vehicle (−) or with Bg 20 μM or Bg 50 μM for 16 h. **b** MCF-7 and ZR75 cells treated with vehicle (−) or with Bg 20 μM or Bg 50 μM for 16 h, were incubated with acridine orange and fluorescence was analyzed by flow cytometry. **c** MCF-7 transfected with non specific (NS) or targeted against NF-Y siRNA treated with vehicle (−) or with Bg 20 μM for 16 h. Images show the results of one representative experiment out of three

We next studied the pattern of subcellular localization of LC3 which re-localizes from the microtubules to autophagosomal membranes. MCF-7 cells were transiently transfected with an LC3-GFP expression vector and subjected to fluorescence microscopy. After 16 h treatment, Bg induced LC3-GFP re-localization and concentration into prototypical autophagic puncta (Fig. 4a).

The autophagic process was also monitored through acridine orange assay that labels acidic vesicular organelles (AVOs) bright red. MCF-7 and ZR-75, treated for 16 h with 20 and 50 μM Bg were stained with acridine orange and analyzed through FACS. As shown in Fig. 4b in MCF-7 cells the red fluorescence increased from 0.35 % (control) to 8.5 % (20 μM Bg) and 44.5 % (50 μM Bg). Analogously, in ZR-75 cells (Fig. 4b) the percentage of red positive cells increased from 1.42 % (control) to 7.7 % (20 μM Bg) and 29.2 % (50 μM Bg) respectively indicating AVO accumulation. To validate the involvement of NF-Y on autophagic phenotype, MCF-7 cells were treated with a specific NF-Y siRNA which substantially reduced the percentage of red positive cells after Bg exposure compared to MCF-7 transfected with NS siRNA (Fig. 4c).

**Fig. 5:**
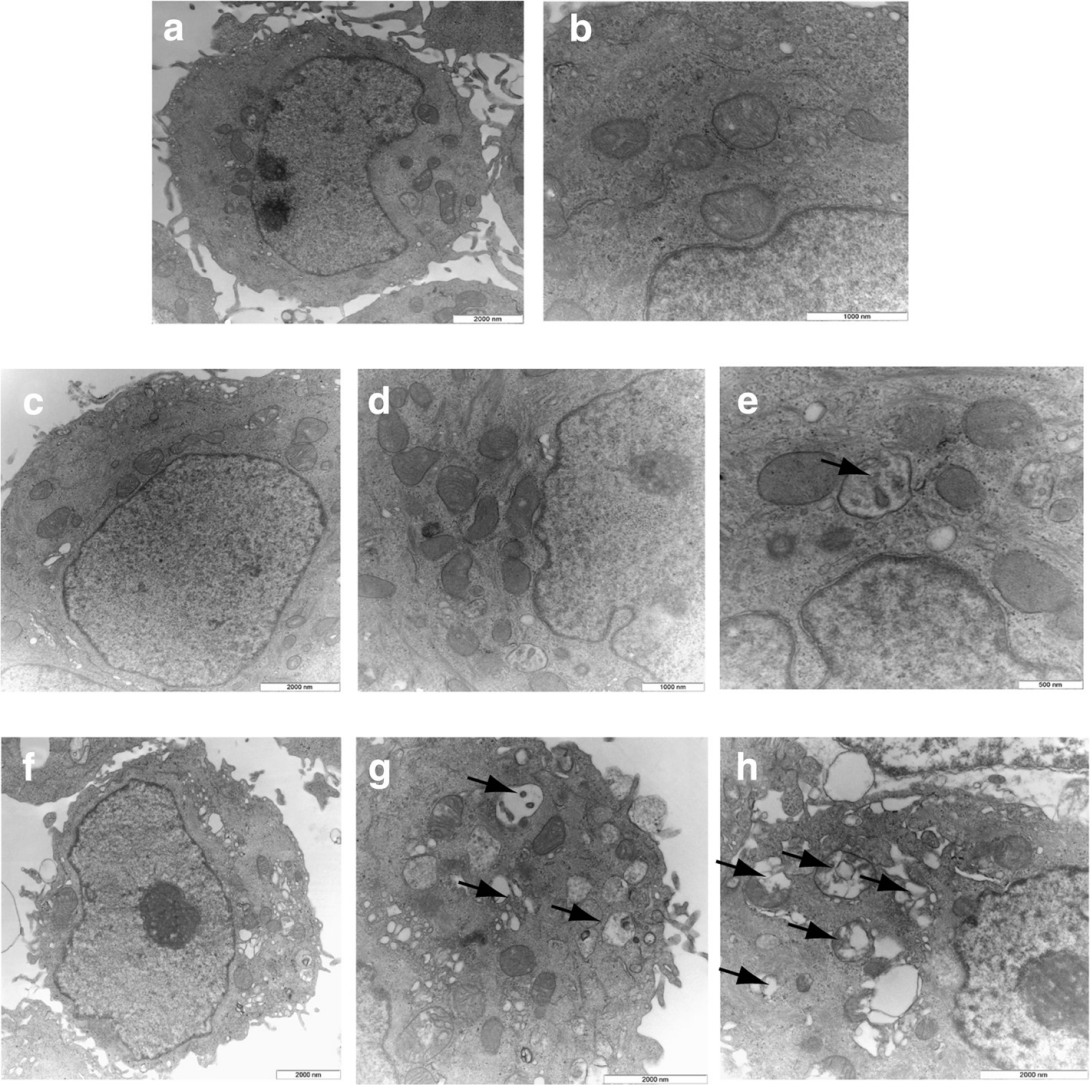
Bergapten treatement evokes an autophagic phenotype in MCF-7. TEM analysis, top panels: untreated MCF7 cells (**a**, **b**). Bottom panels: Bg 20 μM (**c**, **d**, **e**) and Bg 50 μM (**f**, **g**, **H**) treated cells. Morphologic findings characteristic of autophagy are enlarged to highlight the autophagic bodies indicated by the arrows (original magnification: × 10,000). The figure shows the results of one representative experiment repeated at least three times

To further investigate the occurrence of a Bg -dependent autophagic phenotype we used transmission electron microscopy (TEM). As shown in Fig. 5, control cells exhibited normal nuclei with uniform and finely dispersed chromatin, surrounded by cytoplasm with normal appearing mitochondria (panels a, b). 20 μM Bg -treatment for 16 h resulted in the accumulation of autophagic vacuoles (panels c, d and e). Particularly, autophagy was clearly evidenced at 50 μM Bg treated for 16 h (panels f, g, h). Similar results, although in a lesser extent were obtained for ZR-75 cells (data not shown).

### Autophagy favors the inhibition of breast cancer cell survival after Bergapten stimulus

**Fig. 6:**
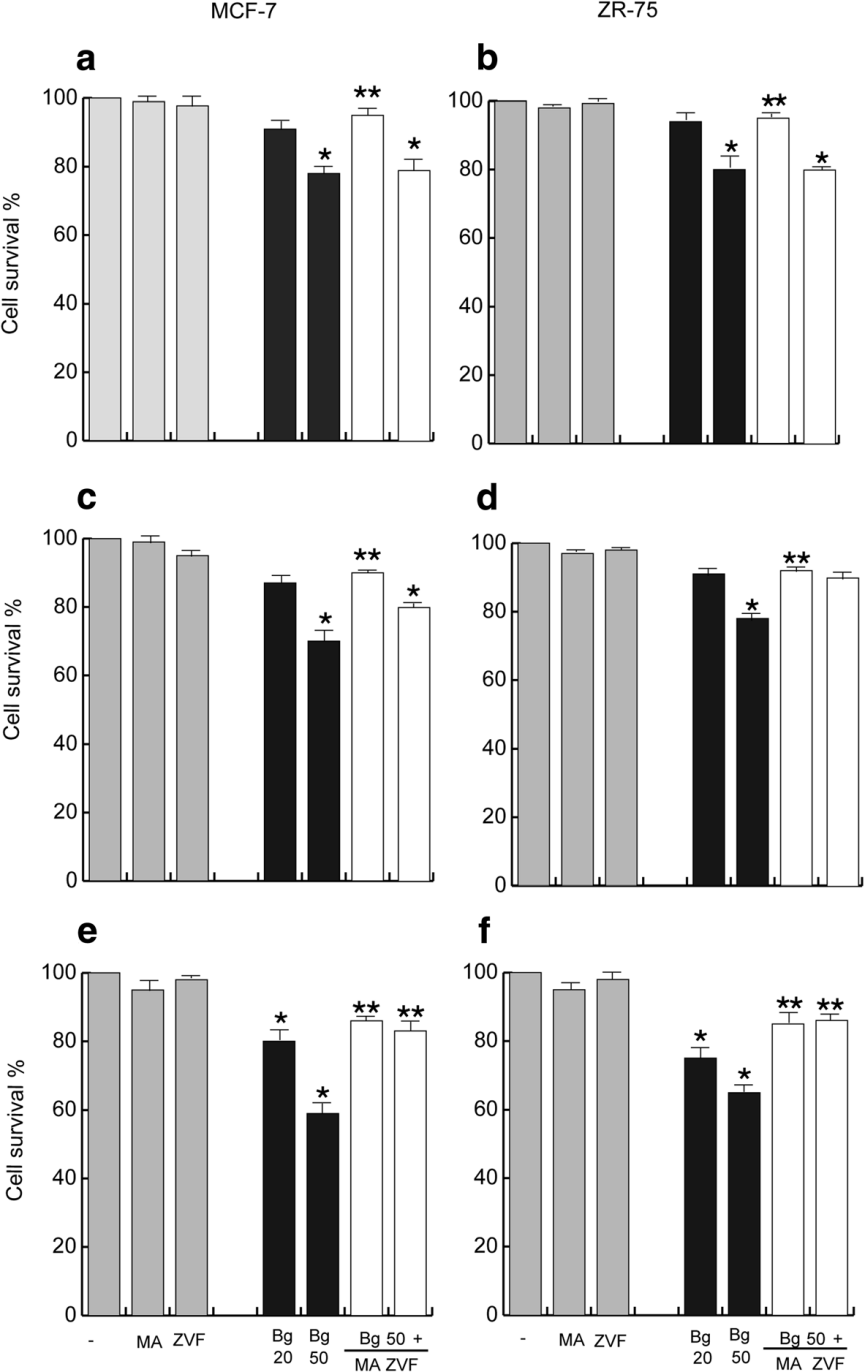
Bergapten treatment reduces cell viability in MCF-7 and ZR-75 cells. MTT assay in MCF-7 and ZR-75 cells treated for 16 h (panel **a**, **b**), 24 h (panel **c**, **d**), 48 h (panel **e**, **f**) with vehicle (−), Bg 20 μM, Bg 50 μM, or pre-treated for 4 h respectively with 5 mM 3-MA (3-Methiladenine) or 10 μM ZVF (Z-VAD-fmk) as indicated. Columns are mean of three independent experiments and expressed as % over vehicle, which was assumed to be 100 %; bars, SD; *P < 0.05 vs vehicle. ** *P* < 0.05 vs Bg 50 μM

To comprehend if the autophagic phenotype evoked by Bg treatment could favor or delay breast cancer cell death, we analyzed the influence of autophagy on cell survival after different times of Bg addition. Our results show that both MCF-7 and ZR-75 cells exposed to Bg exhibited a significant loss of cell survival (Fig. 6), especially under Bg 50 μM. Interestingly co-treatment with the autophagy inhibitor 3-MA effectively counteracted these inhibitory effects clearly suggesting that autophagy could early determine the inhibition of cell viability due to Bg stimulus. Cotreatment with the caspase inhibitor ZVF was ineffective at 16 h, while it counteracted the Bg action at 48 h of stimulation, indicating that apoptosis influences cell viability later respect to autophagy.

We also observed that pre-treatment of MCF-7 cells with the autophagy inhibitor 3-MA counteracted the appearance of DNA laddering after 24 h of Bg stimulus (data not shown).

The results, collectively, evidence that Bg activates the autophagy which supports the decline of cell survival through the later occurrence of apoptosis.

## Discussion

The importance of the biological function of PTEN rises from its frequent mutations and deletions in human cancer. PTEN governs a plethora of cellular processes including survival, proliferation, and energy metabolism, by suppressing the PI3K/AKT/mTOR pathway [15–17]. Herein, we provided evidences that these molecular signals may be target of the natural product Bg, a psoralen compound that belongs to a group of furanocoumarins, which are found in a variety of fruits and vegetables. On the basis of our previous data demonstrating that Bg in breast cancer cells is able to negatively affect the PI3K/AKT signaling, here we found that this may occur through PTEN, the main negative regulator of this pathway. Our study addresses how Bg, by inducing PTEN expression, produces autophagy in breast cancer cells. Thus we propose that PTEN might be one of the signaling protein through which Bg acts to affect breast cancer cell survival.

For instance this emerges from our data showing that the silencing of this gene was able to abrogate the down-regulation of p-AKT/p-mTOR triggering autophagic process under Bg exposure.

It is worth to mention how the lowering of AKT and mTOR protein levels just rely on the degradative process of proteins along with autophagy.

We deepen the molecular mechanism through which Bg might bring to PTEN elevation by performing transient transfection experiments with different PTEN promoter constructs, showing that the stimulatory effect induced by Bg on this gene was mediated by a region containing NF-Y binding site. In fact, site-direct mutagenesis of this element completely reversed the Bg induced up-regulation of PTEN promoter activity. Furthermore NF-Y silencing counteracted Bg effects on PTEN expression.

Whereas in our previous paper we have shown that the transcription factor NF-Y mediates Bg action, herein we further confirmed the crucial role of the activation of NF-Y, via p38MAPK signaling as the main axis through which Bg is able to maintain the PTEN promoter transactivation as well as mRNA and protein increase. NF-Y regulates transcription of various genes related to the cell cycle and various human diseases, as it is required for the recruitment of RNA polymerase II to permit transcriptional activation [18].

Besides, recent studies reported that p38-dependent stress response can drive the autophagy process [19–21]. Interestingly we evidenced the appearance of an authophagic phenotype by Bg treatment as revealed by fluorescence microscopy of LC3-GFP in breast cancer cells together with an increase of different key hallmarks of the autophagic process such as Beclin 1, PI3K III, UVRAG and Ambra1. Moreover our results reveal lipidation of LC3 and the reduction of p62 along with the activation of autophagy. Both markers further change after 24–48 h of Bg stimulus addressing the incipient appearance of apoptosis [8, 9].

Thus the biological outcome of the functional axis Bg/p38MAPK/NF-Y/PTEN is represented by the appearance of an early autophagic phenotype which is crucial for the later inhibition of breast cancer cell survival since it appears effectively counteracted in the presence of a specific autophagy inhibitor 3-MA in both cell types. We retain that Bg induced autophagy represents a preliminary event that pushes breast cancer cells towards apoptosis as late response, as evidenced by the effects of the caspase inhibitor ZVF which counteracted the inhibitory action of Bg on cell viability but subsequently to 24 h of treatment [8]. In other words the present paper highlights how, in a shorter time, Bg triggering autophagy may create the susceptibility to cell death. For instance, some types of cancers exhibit autophagic changes after treatments with irradiation and chemotherapic drugs [22–26] as adaptative response to protect organisms during periods of enhanced cellular distress.

Several phytoproducts such as the coumarins, have shown to inhibit cancer cell proliferation inducing apoptotic cell death [27, 28]. Furthermore, in vitro exposure of cancer cells to natural products resveratrol and the B-group soya-saponins addresses autophagic process [29–31] and in the human cervical carcinoma cell line it has been reported that the furanocoumarin imperatorin, alone or in combination with cisplatin, is mainly an autophagy inducer in these cells [32].

Whether autophagy is a death-induced mechanism or a protective effort for cellular survival is still a controversy [33, 34]. Most likely, depending on cellular type, functional status, microenvironment and therapeutic agents, the persistence over time of autophagy with the increases of cellular self-degradation, could promote apoptosis.

## Conclusions

Thus, on the basis of our data, we can suggest that autophagy precedes cell death demonstrating that p38MAPK/NF-Y/PTEN represents the signaling pathway driving the appearance of the morpho-functional features of autophagy under Bg stimulus. These effects are prevented in the presence of specific NF-Y siRNA as monitored by flow-cytometry in MCF-7 cells.

In summary, our results contribute to identify PTEN as a key target of Bg action in breast cancer cells for the control of survival and autophagy. PTEN is transcriptionally regulated by Bg through the involvement of NF-Y transcription factor. Treatment of cells enhances the expression of Beclin1 and PI3K III, pivotal factors in the formation of a lipid kinase complex which in turn allow the recruitment and shuttling of UVRAG and AMBRA, thus contributing to the generation of the phagophore. These findings add further insights on the mechanism of action of Bg and address how dietary phytochemical compounds may be implemented in the novel strategies for breast cancer treatment.

## Materials and methods

### Materials

5-Methoxypsoralen or Bergapten (Bg), aprotinin, leupeptin, phenylmethylsulfonyl fluoride (PMFS), sodium orthovanadate, the known inhibitor of autophagy 3-Methyladenine (3MA) were purchased from Sigma Chemical (Milan, Italy). The specific caspases inhibitor Z-VAD-FMK (ZVF) was from R&D Systems (Milan, Italy). Antibodies used in this study were from Santa Cruz Biotechnology (Santa Cruz, CA) except SQSTM1/p62 and Phospho­mTOR (Ser2448) from Cell Signalling (Danvers, MA).

### Plasmids

The firefly luciferase reporter plasmid containing the full-length of the PTEN promoter region pGL3-2768 (−2927/-160) and the different deletion constructs pGL3-612 (−1389/-778), pGL3-341 (−1118/-778), pGL3-139 (−916/-778) gifts from Prof. Xi-Liang Zha, (Shanghai Medical College, Fudan University, Shanghai) [12, 13]. The Renilla luciferase expression vector pRL-TK (Promega, Milan, Italy) was used as a transfection standard. GFP-LC3 Expression Vector #CBA-401 was from Cell Biolabs, Inc. (San Diego, CA).

### Cell culture

Authenticated human breast cancer cells MCF-7 and ZR75-1 (ZR-75) (American Type Culture Collection-ATCC) were acquired in 2010, stored according to supplier’s instructions and used within 4 months after frozen aliquots resuscitations. Both cell lines were maintained in DMEM/F-12 medium containing 5 % fetal calf serum (5 %FCS), 1 % L-glutamine, 1 % Eagle’s nonessential amino acids and 1 mg/ml penicillin/streptomycin in a 5 % CO2 humidified atmosphere. Cells were cultured in phenol red-free DMEM, 0.5 % BSA and 2 mM L-glutamine (serum-free medium), for 24 h (h) before each experiment. Bg stimulation was performed in DMEM/F12 containing 5 % charcoal-treated fetal calf serum. MCF-7 cells express endogenous PTEN, while ZR-75 cells contain a hemizygous deletion of PTEN and a missense mutation in the other allele [35]. The Bg concentrations were chosen on the basis of our previous studies [8, 9].

### Western blotting

Total protein extracts were obtained as previously described [36]. Proteins were resolved on a 10 % sodium dodecyl sulfate–polyacrylamide gel, transferred to a nitrocellulose membrane, probed overnight at 4 °C with the indicated antibodies. β-actin was used as loading control.

### Reverse transcription and real-time PCR

Cells were treated as indicated and processed as described [37] The primers were: 5′CCACCACAGCTAGAACTTATC3′ (PTEN forward); 5′ATCTGCACGCTCTATA CTGC3′ (PTEN reverse); 5′-GGCGTCCCCCAACTTCTTA-3′ (18S forward) and 5′-GGGCATCACAGACCTGTTATT-3′ (18S reverse).

### Transfections and luciferase assays

Transfections were done as previously described [38] using Fugene 6 reagent (Roche Diagnostics, Milan, Italy). Luciferase activity was measured with the Dual Luciferase kit (Promega, Milan, Italy).

### Site-directed mutagenesis

Mutagenesis was performed on pGL3-612 (−1389/-778) of the PTEN promoter by PCR. The sequence for the sense primer was: 5′-ACGACCCCATCTCAGCTTagtcCATCAGTCCTCCACC-3′. The amplified DNA fragment was digested and ligated into pGL3-basic vector. Mutation was confirmed by DNA sequencing.

### Lipid-mediated transfection of siRNA Duplexes

RNA oligonucleotide nonspecific (NS) or directed against PTEN and NF-Y were purchased from Invitrogen (Paisley, UK). Cells were transfected using Lipofectamine 2000 reagent (Invitrogen, Paisley, UK) as previously reported [38] according to the manufacturer’s instructions and then treated as indicated.

### MTT assay

Cells (3 × 10^4^ cells/mL) were plated in 24-well plates and serum-starved for 24 h before the addition of treatment. The MTT assay was performed as briefly described : 100 μL of MTT (2 mg/mL) (Sigma Aldrich, Milan, Italy) were added to each well, and the plates were incubated for 2 h at 37 °C. Then, 500 μL of DMSO were added. The absorbance was measured with the Ultrospec 2100 Prospectrophotometer (Amersham-Biosciences, Milan, Italy) at 570 nm.

### GFP-LC3 fluorescence

LC3 translocation was detected using the green fluorescent protein (GFP)-fused LC3 construct (Cell Biolabs, Inc. San Diego, CA). Briefly, cells were seeded in 6 well plates contained glass coverslips and allowed to attach overnight. 7 μg of LC3-GFP expression plasmid were transfected using Fugene 6 reagent (Roche Diagnostics, Milan, Italy). 6 h after transfection, the cells were treated with vehicle or 20 μΜ or 50 μΜ Bg for 16 h. The coverslips with attached cells were stained with the blue-fluorescent DAPI for nuclear stain. The excess buffer was removed and the coverslips were mounted. Fluorescence analysis was carried out on an OLYMPUS BX51 microscope. Images are representative of three different experiments.

### FACS detection of acidic vesicular organelles

Acidic vesicular organelles (AVOs) were detected and quantified after vital staining with acridine orange to monitor autophagic phenotype [24]. Following treatment, cells were stained with 0.5 μg/ml acridine orange for 15 min at 37 °C. Cells were then trypsinized and collected in phenol-red free medium. Green (510–530 nm) and red (>650 nm) fluorescence emission from cells was measured with a Fluorescence Activated Cell Sorter (FACS) using CellQuest software.

### Transmission Electron Microscopy (TEM)

TEM was conducted as previously described [39] Cells were fixed in 3 % glutaraldehyde solution in 0.1 M phosphate buffer (pH. 7.4) for 2 h. Then the samples were post-fixed in osmium tetroxide (3 %), dehydrated in graded acetone and embedded in Araldite (Fluka, Buchs, Switzerland). Ultrathin sections were collected on copper grids and contrasted using both lead citrate and uranyl acetate. The grids were examined in a “Zeiss EM 10” electron microscope.

### Statistical analysis

Data were analyzed by Student’s *t* test using the GraphPad Prism 4 software program.

## Additional file

Additional file 1: Figure S1. (A) Time course study. Western blot analysis of p-p38 and p38 expression in MCF-7 and ZR-75 cells treated as indicated with vehicle (−), Bg 20 μM, Bg 50 μM. Autoradiographs show the results of one representative experiment out of three. (B) Western blot analysis of PTEN in MCF-7 treated with vehicle (−), Bg 20 μM and/or p38MAPK inhibitor SB203580 (SB 10 μM).
