# Supplementary figures and images for: Bergapten drives autophagy through the up-regulation of PTEN expression in breast cancer cells
Source: Mol Cancer. 2015 Jul 7;14:130. doi: 10.1186/s12943-015-0403-4 (PMC4498523; doi:10.1186/s12943-015-0403-4)

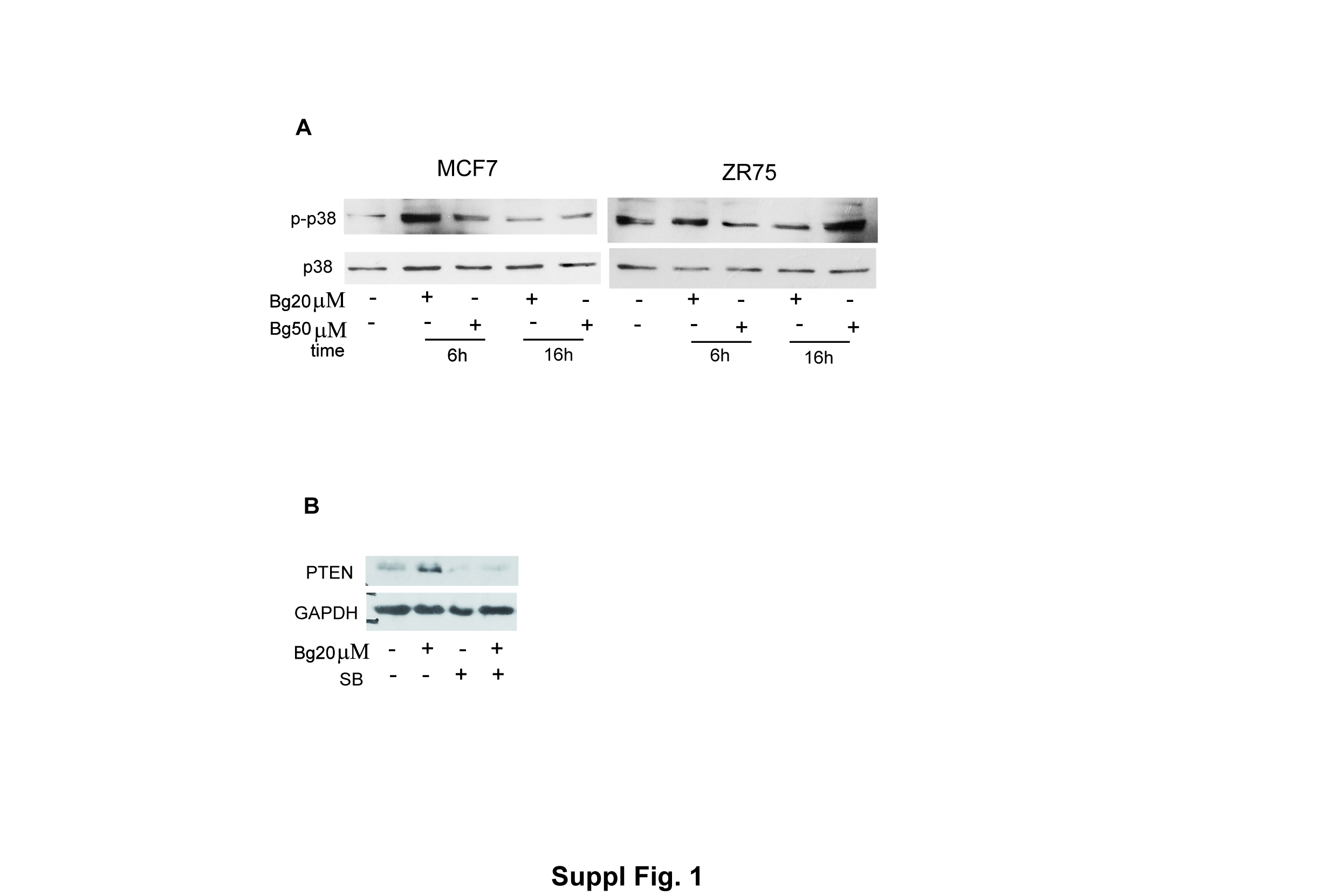

Supplement: Additional file 1: Figure S1. — (A) Time course study. Western blot analysis of p-p38 and p38 expression in MCF-7 and ZR-75 cells treated as indicated with vehicle (−), Bg 20 μM, Bg 50 μM. Autoradiographs show the results of one representative experiment out of three. (B) Western blot analysis of PTEN in MCF-7 treated with vehicle (−), Bg 20 μM and/or p38MAPK inhibitor SB203580 (SB 10 μM). [file 12943_2015_403_MOESM1_ESM.tiff]
